# A parasitological survey of zoonotic cestodes carried by house rats in Aswan, Egypt, reveals cryptic diversity at the molecular level

**DOI:** 10.14202/vetworld.2021.2160-2169

**Published:** 2021-08-23

**Authors:** Abuelhassan Elshazly Younis, Atef Ibrahim Saad, Islam Refaat Mohamed El-Akhal, Nagla Mustafa Kamel Saleh

**Affiliations:** Department of Zoology, Faculty of Science, Aswan University, Aswan 81528, Egypt

**Keywords:** cestodes, cryptic diversity, molecular identification, rat

## Abstract

**Background and Aim:**

Some rat cestodes are zoonotic and are capable of parasitizing humans and animals, raising serious concerns regarding human and veterinary health. The study aimed to determine the prevalence and risk factors for cestodes in Egyptian house rats and to characterize the cestodes molecularly.

**Materials and Methods:**

The current survey examined 115 house rats (*Rattus rattus*) in two cities (Edfu and Aswan) in Egypt’s Aswan Governorate for cestode infection using integrated molecular approaches (polymerase chain reaction, sequencing, and phylogenetic analysis) and morphological/morphometrical approaches.

**Results:**

The cestodes identified in this study exhibited the typical morphological characteristics of *Hymenolepis diminuta* (Rudolphi, 1819), *Hymenolepis nana* (Siebold, 1852) (from rat intestine), and *Hydatigera taeniaeformis* (from rat liver). The species prevalence rates from these three studies were reported to be 8.7%, 10.4%, and 20.9%, respectively. The ribosomal DNA (ITS1, 18S, and complete ITS) sequences revealed that the hymenolepid sequences were highly distinct but were related to other sequences in the GenBank database, with some sequences showing high similarities to those of *H. nana* and *H. diminuta*. In addition, the *H. taeniaeformis* sequences (ITS2 and mitochondrial cytochrome c oxidase subunit 1 [mtCOX1]) obtained in this study were highly similar to some *Taenia taeniaeformis* GenBank sequences. The constructed phylogram revealed that the hymenolepidid tapeworms examined in this study were classified into four major branches (the majority of which were hybrids of the two species) and belonged to the genus *Hymenolepis*. In addition, the phylogram of *H. taeniaeformis* assigned this species to *T. taeniaeformis*.

**Conclusion:**

When typical hymenolepid morphology is combined with molecular and phylogenetic divergence, it may indicate the existence of possible cryptic species. In addition, on the basis of the phylogenetic analysis, genetic diversity within *T. taeniaeformis* may exist as determined by comparing the metacestode mtCOX1 sequences. The current study presents the prevalence values of zoonotic cestodes and contributes to the body of knowledge, including identification keys and the use of molecular tools for species confirmation.

## Introduction

Around 25% of the world’s population may be infected with transmissible parasitic infections, which are typically prevalent in tropical and subtropical regions. Such infections lead to reduced worker productivity and the depletion of economic resources [1]. Rodent-borne helminthiases are neglected diseases that disproportionately affect (but are not limited to) residents of low- and middle-income countries. These cause a variety of health problems in humans, including malnutrition, increased prevalence of malaria and HIV/AIDS, decreased vaccine effectiveness, and increased incidence of allergies [2].

The house rat, *Rattus* *rattus*, is one of the world’s most widespread mammals [3]. It is regarded as a serious pest in urban and rural areas due to the economic damage it causes and the fact that it harbors and spreads zoonotic pathogens such as viruses, bacteria, protozoa, and helminths [4-6].

Rodents can serve as intermediate hosts for cestode parasites and can transmit them to other animals such as cats, in the case of the parasite *Hydatigera taeniaeformis* (*Taenia taeniaeformis*), or as reservoir hosts, in the case of *Hymenolepis* spp. [7]. Hymenolepiasis is a zoonotic parasitic infection spread by the cestodes *Hymenolepis nana* and *Hymenolepis diminuta*. Of these, *H. nana* is by far the most prevalent because it is frequently transmitted directly to children through contaminated hands, dust, food, and water. Human infections with *H. diminuta* are uncommon and typically result from accidental ingestion of small arthropod intermediate hosts. The family Hymenolepididae contains over 920 species of tapeworms that infect birds and mammals [8].

*H. taeniaeformis* is the larval stage of the adult tapeworm *T. taeniaeformis* (alternative names: *Cysticercus fasciolaris*, *Hydatigera fasciolaris*, *Strobilocercus fasciolaris*, or *T. crassicolis*), which is found worldwide and primarily infects the small intestines of felines and canines following the ingestion of infected rodent livers [9]. *H. taeniaeformis* is a potential zoonotic parasite. Although this cestode can infect humans, this is a rare occurrence and has only been reported in individuals from Argentina, the Czech Republic, Denmark, Taiwan, and Sri Lanka [10-12].

Molecular biology techniques such as polymerase chain reaction (PCR) and sequencing can easily identify parasites [13,14]. However, the taxonomic and systematic statuses of Hymenolepididae and Taeniidae, as well as their family and generic phylogenetic relationships, remain unknown [15]. At present, the use of nuclear ribosomal DNA (rDNA) and mitochondrial cytochrome c oxidase subunit 1 (mtCOX1) as markers makes these methods powerful tools for resolving exceptional taxonomic challenges and separating closely related genera and species [16].

Recognizing cestode distribution and population genetics is critical for controlling them since it influences their systematics, epidemiology, genesis, and ecology [17]. The house rat likewise plays a critical role in understanding the epidemiology of diseases transmitted by cestode hosts. Inadequate urban infrastructure and sanitation significantly affect rat abundance and increase the rate of rat–human contact. Thus, residents of underprivileged urban areas are more susceptible to rat-borne infections, with drug users, the immunocompromised, and the homeless being the most disadvantaged [4,18,19]. However, few studies on rodent polyparasitism have been conducted in Egypt, particularly in the Upper Egypt region. There is currently no information on rodent tapeworms in Egypt’s Aswan Governorate, an ancient region of international tourist importance that serves as the country’s southernmost region and gateway to Africa.

This study aimed to conduct a prevalence survey in two locations (Edfu and Aswan) in the Aswan Governorate to ascertain the presence of cestode parasites in house rats and the effect of house rats on cestode parasite transmission. We additionally performed molecular and phylogenetic analyses on nuclear and mitochondrial loci to validate our identifications, examine the rates of genetic variation and differentiation, and augment the cestode genetic dataset.

## Materials and Methods

### Ethical approval

The current study was approved by Aswan University’s Research and Graduate Studies Council. It follows a standard operating procedure that has been approved by Aswan University’s Animal Use and Care Committee.

### Study period and area

**Figure-1:**
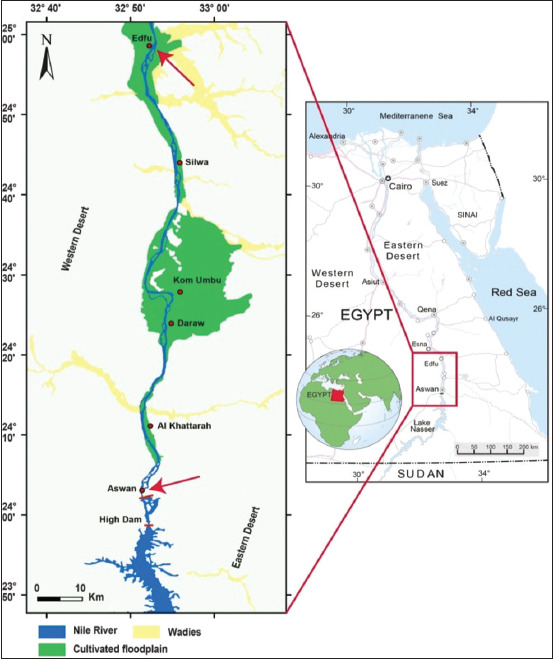
General map of Egypt illustrating the Aswan Governorate and the locations of sampling sites marked by arrows; blue indicates the Nile, green indicates agricultural flood plains, and yellow indicates wadies. Map is modified after [20].

The study was conducted from January 2016 to December 2019 in the residential communities of Aswan and Edfu in Egypt’s Governorate of Aswan. Aswan Governorate is the southernmost governorate in Upper Egypt, with Aswan as its capital city. At latitude 22 north of tropical cancer, Aswan Governorate borders Luxor Governorate to the north, Red Sea Governorate to the east, New Valley Governorate to the west, and Northern State of Sudan to the south. This covers an area of 62,726 square kilometers. Edfu, Aswan’s northernmost city, is located on the border with Luxor, while Aswan, Egypt’s southernmost city, and is located on the Sudanese border (Figure-1) [20]. The two neighborhoods are located in a rural/urban area with a middle socioeconomic status and are defined by paved streets, a variety of small businesses, middle-class housing, and vacant lots. In these neighborhoods, pets (i.e., dogs and cats), weeds, shrubs, and trees are common.

### Animals and parasites

Wire traps were baited with baked cheese and distributed throughout the yard near areas where rodent activity was observed. Fifty traps were set daily for 3 days at each trapping site, comprising approximately 2 km of land. A total of 115 house rats (45 in Edfu and 70 in Aswan) were captured and identified in the field using a morphological rodent identification key [21] and immediately transported to the laboratory. This study used only house rats, and any other species that were captured were released. Rats were then anesthetized and killed in a small container of ether. The rats were dissected immediately after killing, and their internal organs were immersed in physiological saline and examined for tapeworm infections. Any tapeworms found were extracted from the infected rats, washed with saline, and examined under a light stereomicroscope for initial identification. Individual tapeworm fragments were stored at −20°C in 70% ethanol for molecular experiments. The remainder of each tapeworm was fixed in neutral buffered formalin for further morphological confirmation.

### Examination of morphology

Each rat’s isolated tapeworm species were counted to determine the prevalence and intensity of infection. Mature eggs of Hymenolepididae were obtained from terminal gravid proglottids in saline solution. The fixed cestode parasites were then sequentially fixed, stained with alum carmine, dehydrated in ascending grades of alcohol, cleared in xylene, and finally mounted in Dibutylphthalate Polystyrene Xylene. The tapeworms were then morphologically and morphometrically examined using a light microscope (Olympus CX41, Japan) and photographed using a digital camera (Olympus DP74) attached to the microscope (Olympus BX43). Drawings were created using a drawing tube, and measurements in millimeters were taken using a light microscope. The tapeworms were then identified using morphological taxonomic Key [22].

### Molecular analysis

#### Genomic DNA (gDNA) extraction

gDNA was isolated from individual ethanol-preserved tapeworms using a slightly adapted phenol/chloroform protocol [23]. In brief, the parasite materials were digested with proteinase K in ALT buffer overnight at 56°C (Dneasy Kit, Qiagen) and then precipitated with 5.2 M ammonium acetate. The samples were diluted in 20-50 μL of dH_2_O according to pellet size, and concentrations of gDNA samples were determined spectrophotometrically using a Nanodrop spectrophotometer (Implen NP80, Germany).

#### Primer design and PCR

The following markers and primers were used:

A.*Hymenolepis* spp.
  1.Ribosomal ITS1 gene using the forward BD1 (5′-GTCGTAACAAGGTTTCCGTA-3′) and reverse 4S (5′-TCTAGATG CGTTCGAAGTGTCGATG-3′) primers [24]
  2.Entire ITS sequence using the forward P1 (5′-GTCGTAACAAGGTTTCCGTAGGTG-3′) and reverse P2 (5′-TATGCTTAAGTTCAGCGGGTAATC-3′) primers [25]
  3.Ribosomal 18S marker using the forward and reverse primers 18S81 (5′-TTCACCTACGGAAACCTTGTTACG-3′) and 18S83 (5′-GATACCGTCCTAGTTCTGACCA-3′) [26].
B.*H. taeniaeformis*
  1.For ITS1, two pairs of primers were used: Forward MCesS1F (5′- GCGTGTCC GTCTCTCTCT-3′) and reverse MCesS1R (5′-ACGCACA ACCATCACCACTA-3′). These were designed using GenBank accession number FJ939134. These were followed by the forward CesS1F (5′-GCCCGAGAGGAGTTGTGTTA-3′) and reverse CesS1R (5′-AAGGGA GAAGGGAAG ACCAA-3′) using GenBank accession number EU051351
  2.For ITS2, two pairs of primers were designed: Forward MCesS2F (5′-TGACTTCCATTGCGTCCATA-3′) and reverse MCesS2R (5′-GCACAG CTGACCTGTACTGC-3′) using the GenBank accession number FJ939133. These were followed by the other forward CesS2F (5′-GCACACCCTAACCCAAACAC-3′) and reverse CesS2R (5′-ACGGTG GATAGGGGCTGTAT-3′) using GenBank accession number EU051352
  3.A pair of primers was designed for mitochondrial COX1 using the GenBank accession number FJ939135: forward MCesC1F (5′-AACCCCACCAAACGTAAACA-3′) and reverse MCesC1R (5′-AGGAAGA AGGGTGAGGTC-3′).

PCR amplifications were performed in a total volume of 50 μL containing 1× thermo buffer (MyTaq Red Reaction buffer, Bioline. UK), 10-20 pmol forward primer, 10-20 pmol reverse primer, 10 mM dNTPs mix (Alliance Bio, USA), 5 u/μL Taq polymerase (Bioline, UK), and 0.1-0.2 μg gDNA. The amplifications were performed in a thermocycler (Sensoquest Lab cycler, SensoQuest GmbH, Germany) under the following cycling conditions: Initial denaturation at 95°C for 5 min, followed by 35 cycles at 94°C for 30 s; annealing at 54-58°C for 30 s; elongation at 72°C for 30 s; and final extension at 72°C for 5 min. Samples containing rat intestine DNA or lacking gDNA were included in the PCR as controls. Amplification products were analyzed and stained with ethidium bromide on 1% agarose gel. The DNA bands were then visualized and photographed immediately using an ultraviolet (UV) gel documentation unit (UVP Bio-Doc IT-220 Imaging system, BioExpress, USA).

#### Sequencing

The PCR products were purified according to the manufacturer’s protocol using the DNA Clean and Concentrator Kit (Zymo Research, USA) or QIAquick^®^ PCR Purification Kit (Qiagen, Hilden, Germany). Purified PCR products were sequenced in both directions (forward and reverse) using the same primers as in the initial PCR using a dideoxy termination method (Macrogen Inc., Korea) and an Applied BioSystems sequencer automated DNA sequencing system (Model 3730XL) (Applied BioSystems, USA).

#### Sequence and phylogenetic analysis

The NCBI Blast program was used to conduct homology searches on each sequence (http://www.ncbi.nlm.nih.gov/). Each forward sequence was then compared to its reverse complement and manually adjusted and assembled using the CAP3 program. Following that, the generated sequences were aligned to each other and to the most homologous sequences in the database using the multiple sequence alignment program CLUSTALW. Phylogenetic trees were constructed using the online tool Phylogeny.fr, aligned using MUSCLE (v3.8.31), and optimized for maximum accuracy (MUSCLE with default settings). After alignment, ambiguous regions (i.e., containing gaps and/or misaligned) were removed using Gblocks (v0.91b). The phylogenetic tree was then reconstructed using the PhyML software’s maximum likelihood approach (v3.1/3.0 aLRT). Finally, TreeDyn (v198.3) was used to graphically represent and edit the phylogenetic tree [27].

## Results

### Morphological identification

**Figure-2:**
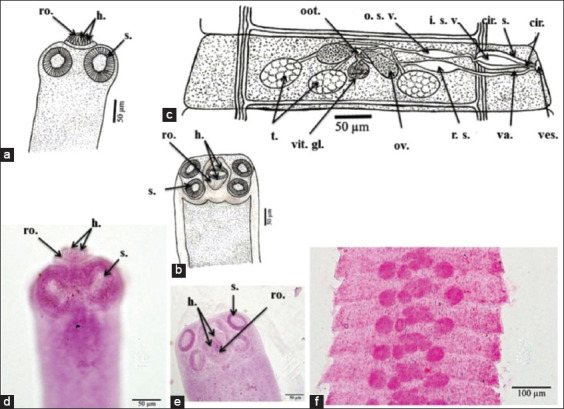
*Hymenolepis nana* morphological characteristics. (a and b) A camera lucida drawing of scolex reveals a retractile rostellum with hooks and suckers; (c) A camera lucida drawing of mature proglottid reveals the structure of the genital organs; (d and e) A microphotograph of scolex reveals a rostellum and suckers; and (f) A microphotograph of mature proglottid. cir.=Cirrus, cir. s.=Cirrus sac/pouch, h.=Hook(s), i. s. v.=Inner seminal vesicle, o. s. v.=Outer seminal vesicle, oot.=Öotype, ov.=Ovary, r. s.=Receptaculum seminis, ro.=Rostellum, s.=Sucker(s), t.=Testis/Testes, u.=Uterus, va.=Vagina, ves.=Vestibule, vit. g.=Vitelline gland(s).

**Figure-3:**
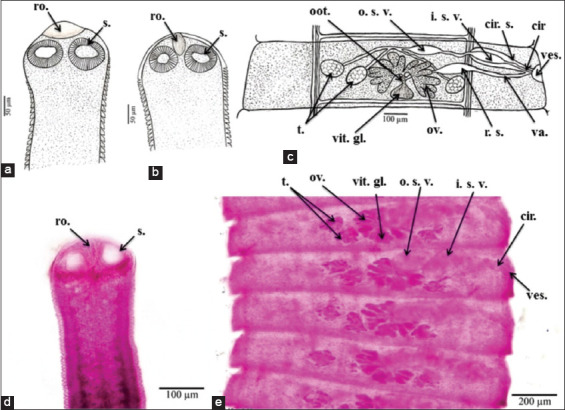
*H. diminuta* morphological characteristics. (a and b) A camera lucida drawing of a scolex reveals the rostellum and suckers; (c) a camera lucida drawing of a mature proglottid reveals the structure of the genital organs; (d) a microphotograph of a scolex reveals the rostellum and suckers; and (e) a microphotograph of a Mature proglottid. cir.= Cirrus, cir. s.=Cirrus sac/pouch, i. s. v.=Inner seminal vesicle, o. s. v.=Outer seminal vesicle, oot.=Öotype, ov.=Ovary, r. s.=Receptaculum seminis, ro.=Rostellum, s.=Sucker(s), t.=Testis/testes, u.=Uterus, va.=Vagina, ves.=Vestibule, vit. g.=Vitelline gland(s).

**Figure-4:**
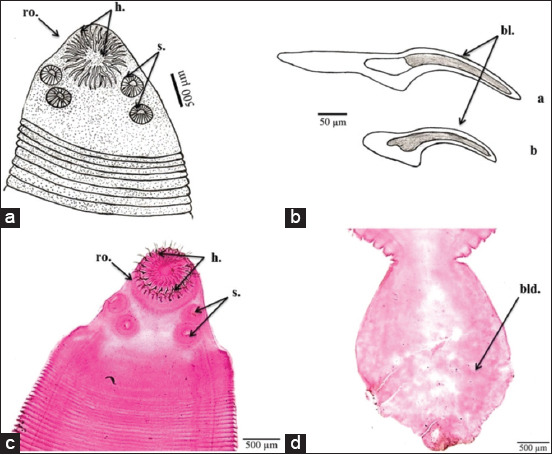
*Hydatigera taeniaeformis* morphological characteristics. (a) Camera lucida drawing of scolex with rostellum and four suckers; (b) camera lucida drawing of outer hooks (a) and inner hooks (b); (c) microphotograph of scolex with rostellum and suckers; and (d) microphotograph of terminal bladder. bl.=Blade, bld.=Bladder, h.=Hook(s), ro.=Rostellum, s.=Sucker(s).

The morphological characters depicted in Figures-2-4 are the result of light microscope examinations and drawings of *H. nana*, *H. diminuta*, and *H. taeniaeformis*.

*H. nana* was identified as 10 small cestodes found in the rat’s small intestinal lumen, and these were creamy white and measured 35 mm (ranging from 27.5 to 42.5 mm) in total body length. The body consisted of a small scolex with an armed rostellum and a multistrobila that was wider than its length. The scolex diameter measured approximately 0.17 (range, 0.12-0.20) mm, whereas the sucker diameter measured approximately 0.07 (0.05-0.08) mm. The rostellum was armed with approximately 23 (22-24) hooks with an approximate length of 0.013 (0.012-0.015) mm. Immature proglottids had an average length of 0.025 (0.020-0.030) mm and an average width of 0.185 (0.170-0.200) mm. Mature proglottids measured approximately 0.085 (0.050-0.120) mm in length and 0.485 (0.170-0.800) mm in width. The male reproductive system consisted of three spherical testes (one poral and two antiporal). The testes measured approximately 0.060 (0.050-0.063) mm in diameter. Bipartite seminal vesicles, one outer and one inner, led to the cirrus and were encircled by a cirrus pouch. The female reproductive system consisted of a bilobed ovary, with each lobe measuring 0.057(0.050-0.063) by 0.055(0.025-0.030) mm in the median of the proglottid and opening into a small öotype receiving a vitelline duct from a large compact vitelline gland. The gravid proglottid had an average length of 0.16 (0.13-0.18) mm and an average width of 0.85 (0.75-1.00) mm. The average egg diameter was 0.040 (0.035-0.050) mm. The average embryo diameter was 0.026 (0.025-0.030) mm (Figure-2).

*H. diminuta* was identified as eight slightly longer cestodes found in the rat’s small intestinal lumen, and these were creamy white and measured approximately 330 mm (ranging from 220 to 450 mm) in total body length. The body consisted of a small scolex and a series of long multistrobila with greater length than width. The scolex diameter measured approximately 0.17 (0.12-0.20) mm, whereas the sucker diameter measured approximately 0.08 (0.06-0.10) mm. Immature proglottids had an average length of 0.055 (0.030-0.070) mm and an average width of 0.75 (0.45-1.35) mm. Mature proglottids measured approximately 0.16 (0.13-0.20) mm in length and 1.35 (1.20-1.50) mm in width. The male reproductive system consisted of three spherical testes (one poral and two antiporal). The testes measured approximately 0.12 (0.10-0.15) mm in diameter. Bipartite seminal vesicles, one outer and one inner, led to the cirrus and were encircled by cirrus pouch. The female reproductive system consisted of a bilobed rosette-shaped ovary, with each lobe measuring 0.165 (0.150-0.180) by 0.115 (0.100-0.130) mm in the median of the proglottid and opening into a small öotype receiving a vitelline duct from a large compact vitelline gland. The gravid proglottid had an average length of 0.45 (0.40-0.55) mm and an average width of 3.4 (2.5-4.0) mm. The average egg diameter was 0.040 (0.035-0.050) mm. The average embryo diameter was 0.026 (0.025-0.030) mm (Figure-3).

*H. taeniaeformis* larvae exhibited typical characteristics of the family Taeniidae. Specimens were extracted from rat livers and were encased in a creamy, fibrous cyst filled with clear and transparent cysticercus fluid. The cysts measured approximately 6.5 (2-10) mm in diameter. The body consisted of a scolex and immature strobila and measured an average of 19 (6.25-31.25) mm in length. The scolex measured approximately 1.6 (1.3-1.7) mm in length and 1.6 (1.2-1.9) mm in width, with four prominent lateral suckers measuring approximately 0.035 (0.030-0.040) mm in diameter. The scolex supplied a rostellum armed with double rows of 34-40 hooks, with the outer row containing large hooks of 0.42(0.38-0.45) mm length and the inner row containing smaller hooks of 0.23 (0.19-0.27) mm length. All of the hooks were taenoid in design, with long, and blunt handles that measured 0.21 (0.18-0.23) mm (large hooks) and 0.11 (0.09-0.12) mm (small hooks). Each hook had a sharply curved pointed blade measuring 0.23 (0.20-0.25) mm (large hooks) and 0.13 (0.10-0.15) mm (small hooks). The strobila consisted of short segments that terminated in a bladder, resembling a small tapeworm but lacking reproductive organs. All of these morphological characteristics were consistent with the larval stage of *T. taeniaeformis* (Figure-4).

### Prevalence and distribution

In our survey, we identified 115 rats as *R. rattus* based on morphological characteristics. Of these, 42 rats (36.52%) were found to be infected with cestodes. Two species of hymenolepidid cestodes were identified in the rat intestines, namely, *H. nana* and *H. diminuta*, which were found in 10.43% and 8.70%, respectively, of the total number of rats. We additionally detected the taeniid *H. taeniaeformis* cyst stage in 20.87% of the examined rat livers (Table-1).

**Table-1:** Cestodes prevalence and mean intensity.

Helminthes	Frequency	Prevalence %	No. of Parasite in each rat	Mean Intensity
Cestodes	42	36.52		
*Hymenolepis diminuta*	10	8.70	1-4	0.1-0.5
*Hymenolepis nana*	12	10.43	1-5	0.09-0.5
*Hydatigera taeniaeformis*	24	20.87	1-25	0.05-1.13

Table-2 shows the distribution of cestode infection in house rats for two sites in the Aswan Governorate. All three detected species were discovered at both of the sites investigated. *H. nana* and *H. diminuta* were both prevalent in Edfu City, accounting for more than 57% and 35% of infected rats, respectively, and *H. taeniaeformis* was more prevalent in Aswan than in Edfu. Aside from a few cases where two species coinfected a single rat, the rats were mostly infected by a single species. No rat was found to be coinfected by all three species (Table-3).

**Table-2:** Infection distribution in two locations (Aswan and Edfu cities).

Distribution	Aswan	Percentage	Edfu	Percentage
Examined rats	70	60.87	45	39.13
Infected by cestodes	28	40.00	14	31.11
Uninfected by cestodes	42	60.00	31	68.89
Infected by *Hymenolepis diminuta*	5	17.86	5	35.72
Infected by *Hymenolepis nana*	4	14.29	8	57.14
Infected by *Hydatigera taeniaeformis*	20	71.43	4	28.57

**Table-3:** Infection of rats with single and mixed cestode species.

Diagnosis	No. of infected rats	Percentage
Single infection by *Hymenolepis diminuta*	6	14.29
Single infection by *Hymenolepis nana*	10	23.81
Single infection by *Hydatigera taeniaeformis*	22	52.38
Mixed infection by *Hymenolepis diminuta* and *Hymenolepis nana*	2	4.76
Mixed infection by *Hymenolepis diminuta* and *Hydatigera taeniaeformis*	2	4.76

### Molecular analysis

**Figure-5:**
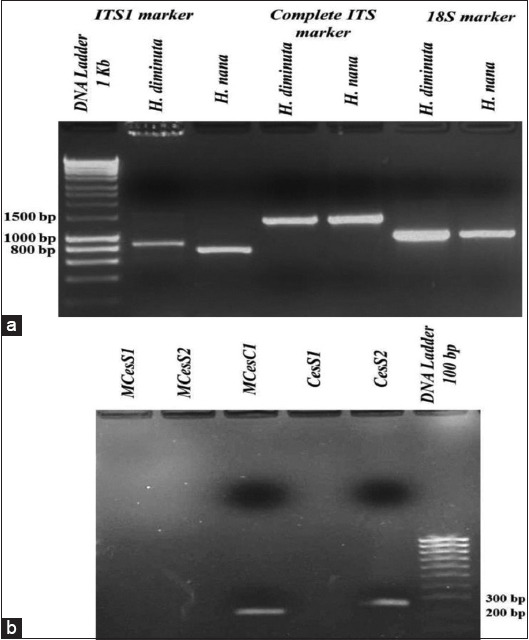
Gel analysis of PCR products from the three detected cestodes species under UV light stained with ethidium bromide. (a) Hymenolepis amplicons for gene markers: ITS1 ~ 900 bp; complete ITS ~ 1500 bp; 18S ~ 1050 bp; and (b) Amplicons of *Hydatigera taeniaeformis* mtCOX1 ~ 200 bp and ITS2 ~ 250 bp.

The ITS1, complete ITS, and 18S amplicons obtained from the gDNA samples of *H. diminuta* and *H. nana* were 900, 1500, and 1050 bp, respectively (Figure-5a). Three *H. diminuta* sequences were generated and deposited into GenBank for validation under the accession numbers MF143798 (ITS1), MF067416 (complete ITS), and MT448710 (18S). Blast analyses of the three sequences revealed that they were distinct from all other sequences in the GenBank databases. The ITS1 and 18S sequences bore no resemblance to other *H. diminuta* sequences. However, the complete ITS sequence had a high identity (92%) to two *H. diminuta* sequences (accession numbers MK787167 and MK787168) in the GenBank databases with 48% query coverage. Interestingly, both the newly submitted ITS1 and the complete ITS sequences demonstrated 100% identity with 54% coverage due to the fact that ITS1 is a fragment of the complete ITS. Three *H. nana* sequences were generated and deposited into GenBank for validation under the accession numbers MT454661 (ITS1), MT448788 (complete ITS), and MT448789 (18S). The three blast analyses revealed these sequences to have 98.2%, 94.19%, and 97.27% identity, respectively, for the closest GenBank database sequences (AF461124, AF461124, and AY193875). Interestingly, the newly submitted ITS1 and complete ITS revealed a 90.93% identity with 68% coverage of each other.

Only one of the designed primer pairs (CesS2F/CesS2R) successfully amplified the *H. taeniaeformis* ITS2 rDNA marker. Amplicons of 250 bp (ITS2) and 200 bp (mitochondrial COX1) were generated (Figure-5b). Two sequences were obtained and submitted to GenBank for validation under the accession numbers MT454664 (ITS2) and MT512661 (COX1). A blast search of the ITS2 sequence revealed 98.84% identity to *T. taeniaeformis* (GenBank accession number FJ939133). Additional blast analyses of the COX1 sequence revealed a high degree of identity (99.53%) to eight *T. taeniaeformis* sequences (GenBank accession numbers MH938573, MH938572, MH036509, AP017671, KT693060, KT693055, KT693054, and AB745096).

**Figure-6:**
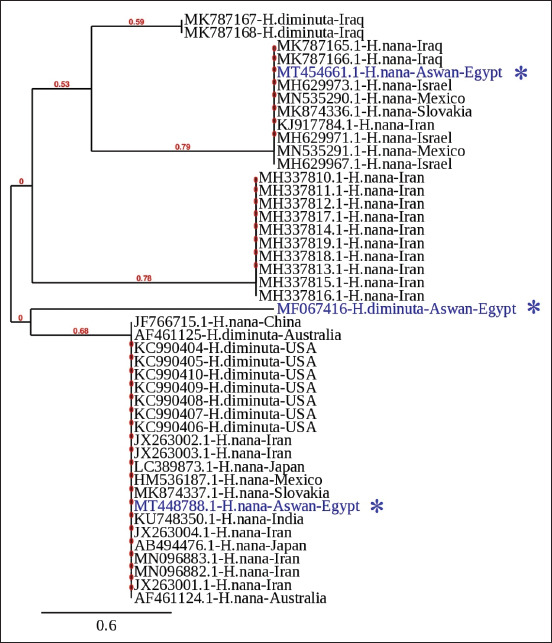
A phylogenetic tree was constructed using the Phylogeny.fr program (http://www.phylogeny.fr/) utilizing the most related and aligned complete ITS sequences of *Hymenolepis nana and Hymenolepis diminuta*, including those identified in this study (*). The branches display the Bootstrap support values. The compared sequences are denoted by accession numbers, names, and locations.

The 45 most relevant nucleotide sequences from *H. diminuta* (complete ITS) and *H. nana* (complete ITS and ITS1) were used in the phylogenetic analyses (Figure-6). A constructed phylogram revealed that the hymenolepidid tapeworms examined in this study comprised four major branches, mostly mixed with both species, with no effect on their geographical distributions. These were found to be embedded within the genus *Hymenolepis* with considerable divergence, particularly in *H. diminuta*.

**Figure-7:**
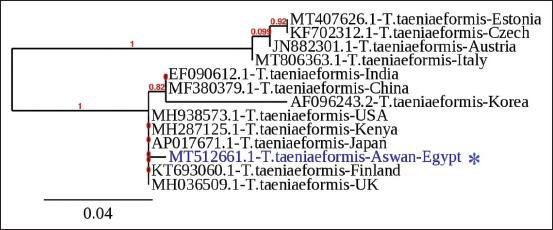
A phylogenetic tree was constructed using the Phylogeny.fr program (http://www.phylogeny.fr/) with the closely related and aligned *Hydatigera taeniaeformis* mtCOX1 sequences, including the sequence identified in this study (*). Bootstrap support values are displayed in the branches. Accession numbers, names, and locations are used to identify the compared sequences.

An additional phylogenetic tree was constructed by comparing the metacestodes mtCOX1 sequences to the most closely related *T. taeniaeformis* sequences obtained from GenBank (Figure-7).

## Discussion

This study is the first to use integrated molecular and morphological approaches to characterize cestodes from house rats (*R. rattus*) in Aswan, Egypt. The cestodes detected in this study were classified as belonging to *H. nana*, *H. diminuta*, and *H. taeniaeformis* of the order Cyclophyllidea. These findings corroborate Owen’s assertion [28] that rat cestodes are all Cyclophyllideans and belong to the Hymenolepididae and Taeniidae families in particular. Our measurements of *H. diminuta* and *H. nana* morphology independently verify Younis’ description [29] and Hughes’ key [30] to *Hymenolepis* species.

We identified two hymenolepid species from rat small intestines in the Aswan Governorate. We found that *H. diminuta* had a similar prevalence in our study(8.70%) as it did in a similar study (11.67%) in which these species were observed in rats of Egypt’s Assiut Governorate [29]. However, our observed *H. diminuta* prevalence was lower than that of [31], who reported *H. diminuta* infection prevalence in 15.3% of *R. rattus*. In addition, *H. nana* had a prevalence of 10.4% in our study, which was similar to the prevalence rate of 13.33% reported in the Assiut Governorate [29].

We detected infection with *H. taeniaeformis* at a prevalence of 20.9%, which is consistent with the previous study in *Rattus* spp. in the Assiut Governorate [29], but higher than the prevalence of 7.8% reported in the same host in Yucatan Mexico [9] and of 7.4% reported in *Rattus* spp. in Iran [32]. On the other hand, the present study detected a lower prevalence of *H. taeniaeformis* in rats than did similar studies in Argentina (39.5%) [33], Korea (33.8%) [34], and Grenada, West Indies (67.6%) [35]. Although the prevalence of helminth infection in rats varied between studies, these variations may be attributed to the differences in climatic conditions, environmental hygiene, previous control interference, socioeconomic status of the occupants, intermediate host abundance, and host vulnerability to parasite infection [2,36].

The morphological characteristics of the present *H. taeniaeformis* specimens are consistent with previous work in Egypt [29] and in India [37], who detected and described metacestode infection in *R. rattus*. We detected pseudosegmentation along the strobila and a small terminal bladder, correlating with [9,38], who reported *H. taeniaeformis* to be the only metacestode in which the scolex is not invaginated at the bladder but rather attached to it through strobilae.

Our molecular analysis revealed that the *H. nana* sequences were distinct but related to other sequences from the same species in the GenBank database, whereas ITS1 and complete ITS showed 98.2% and 94.3% similarity, respectively, to *H. nana* sequences obtained from Australia [39]. In addition, the 18S sequence was 97.3% identical to *H. nana* isolated from a human in London [40]. Although our complete ITS sequence of *H. diminuta* showed 92% similarity to *H. diminuta* obtained from a rodent in Iraq [41], the ITS1 and 18S sequences showed no similarity to other hymenolepid reference sequences in the GenBank database.

Our phylogenetic analyses assigned *H. diminuta* and *H. nana* to four hybrid branches, indicating the presence of possible cryptic species. These findings corroborate those of Nkouawa *et al*. [42], who reported that identifying and diagnosing hymenolepidid tapeworms is challenging, most likely due to the presence of cryptic species.

The metacestode larval stage sequences obtained in this study were highly similar to *T. taeniaeformis* sequences in GenBank, particularly the ITS2 sequence, which was found to be highly similar (98.9%) to the ITS2 sequence of *T. taeniaeformis* obtained from *R. rattus* in India [37]. In addition, the mtCOX1 sequence was found to be highly similar (99.53%) to the COX1 sequence of *T. taeniaeformis* collected from cats in Georgia, USA [43]. Given the phylogenetic tree’s relative branch lengths and the nodal support that connects them, it is possible that genetic diversity exists within *T. taeniaeformis*. This finding corroborates the findings of Lavikainen *et al.*, [44] Jia *et al*. [45], who discovered significant sequence variations in isolate gene markers of *T. taeniaeformis* that are predictive of cryptic species.

The current study confirms that rodents pose a parasitic threat to both human [46,47] and veterinary [48] health, and corroborates Dyab’s report [49] that some rodent-borne parasites were detected in Aswan schoolchildren, including 3% *H. nana* infection.

## Conclusion

This study sheds light on the potential public health and veterinary consequences of house rats in Egypt’s Aswan Governorate. Three zoonotic cestodes were identified and characterized: *H. diminuta*, *H. nana*, and *H. taeniaeformis*, all of which exhibit molecular characteristics consistent with the presence of cryptic species. This study contributes to the narrowing of the knowledge gap on rat-borne helminthiases by providing additional references to identification keys and the use of molecular tools for species confirmation. However, additional research on the dynamics and socio-environmental risk factors of human–rat–helminth infection remains necessary.

## Authors’ Contributions

AEY: Supervisor who contributed to the technique and practical aspects of molecular biology, as well as data analysis and interpretation. AIS: Head supervisor who developed the idea and the study design and contributed to the morphological section’s explanation. This study is a part of IRME’s Master’s thesis, for which he conducted the majority of the practical experiments. NMKS: Assistant supervisor who assisted with the methodology and practical aspects of morphological identification. All authors drafted and revised the manuscript then read and approved the final manuscript.

## References

[1] Reeder M.M, Palmer P.E.S, Margulis A.R, Burhenne H.J (1994). Parasitic disease. Alimentary Tract Radiology.

[2] Gliga D.S, Pisanu B, Walzer C, Desvars-Larrive A (2020). Helminths of urban rats in developed countries:A systematic review to identify research gaps. Parasitol. Res..

[3] Battersby S, Hirschhorn R.B, Amman B.R, Bonnefoy X, Kampen H, Sweeney K (2008). Commensal rodents. Public Health Significance of Urban Pests.

[4] Himsworth C.G, Parsons K.L, Jardine C, Patrick D.M (2013). Rats, cities, people, and pathogens:A systematic review and narrative synthesis of literature regarding the ecology of rat associated zoonoses in urban centers. Vector Borne Zoonotic Dis..

[5] Angley L.P, Combs M, Firth C, Frye M.J, Lipkin I, Richardson J.L, Munshi-South J (2018). Spatial variation in the parasite communities and genomic structure of urban rats in New York city. Zoonoses Public Health.

[6] Strand T.M, Lundkvist Å (2019). Rat-borne diseases at the horizon. A systematic review on infectious agents carried by rats in Europe 1995-2016. Infect. Ecol. Epidemiol.

[7] Galan-Puchades M.T, Sanxis-Furio J, Pascual J, Bueno-Mari R, Franco S, Peracho V, Montalvo T, Fuentes M.V (2018). First survey on zoonotic helminthosis in urban brown rats (*Rattus norvegicus*) in Spain and associated public health considerations. Vet. Parasitol..

[8] Mariaux J, Tkach V.V, Vasileva G.P, Waeschenbach A, Beveridge I, Dimitrova Y.D, Haukisalmi V, Greiman S.E, Littlewood D.T.J, Makarikov A.A, Phillips A.J, Razafiarisolo T, Widmer V, Georgiev B.B, Caira J.N, Jensen K (2017). Cyclophyllidea van Beneden in Braun, 1900. Planetary Biodiversity Inventory (2008-2017):Tapeworms from Vertebrate Bowels of the Earth.

[9] Medina-Pinto R.A, Torres-Castro M.A, Medina-Pinto R.A, Bolio-González M.E, Rodríguez-Vivas R.I (2019). Natural *Cysticercus*
*fasciolaris* infection in rodents from a rural area in Yucatan, Mexico. Vet. Mex..

[10] Miyazaki I (1991). An Illustrated Book of Helminthic Zoonoses.

[11] Ekanayake S, Warnasuriya N.D, Samarakoon P.S, Abewickrama H, Kuruppuarachchi N.D, Dissanaike A.S (1999). An unusual “infection”of a child in Sri Lanka, with *Taenia*
*taeniaeformis* of the cat. Ann. Trop. Med. Parasitol.

[12] Oryan A, Alidadi S (2015). Public health concerns of *Taeniidae* and their metacestodes. Trop. Med. Surg..

[13] Julius R.S, Schwan E.V, Chimimba C.T (2018). Molecular characterization of cosmopolitan and potentially co-invasive helminths of commensal, murid rodents in Gauteng Province, South Africa. Parasitol. Res.

[14] Sricharern W, Inpankaew T, Kaewmongkol S, Jarudecha T, Inthong N (2021). Molecular identification of *Trichuris trichiura* and *Hymenolepis diminuta* in long-tailed macaques (*Macaca fascicularis*) in Lopburi, Thailand. Vet. World.

[15] Haukisalmi V, Hardman L.M, Foronda P, Feliu C, Laakkonen J, Niemimaa J, Lehtonen J.T, Henttonen H (2010). Systematic relationships of hymenolepidid cestodes of rodents and shrews inferred from sequences of 28S ribosomal RNA. Zool. Scr..

[16] Al-Olayan E, Elamin M, Alshehri E, Aloufi A, Alanazi Z, Almayouf M, Bakr L, Abdel-Gaber R (2020). Morphological, molecular, and pathological appraisal of *Hymenolepis*
*nana* (*Hymenolepididae*) infecting laboratory mice (*Mus*
*musculus*). Microsc. Microanal..

[17] Von Nickisch-Rosenegk M, Brown W.M, Boore J.L (2001). Complete sequence of the mitochondrial genome of the tapeworm *Hymenolepis*
*diminuta*:Gene arrangements indicate that *Platyhelminthes* are Eutrochozoans. Mol. Biol. Evol..

[18] McVea D.A, Himsworth C.G, Patrick D.M, Lindsay L.R, Kosoy M, Kerr T (2018). Exposure to rats and rat-associated *Leptospira* and *Bartonella* species among people who use drugs in an impoverished, inner-city neighborhood of Vancouver, Canada. Vector Borne Zoonotic Dis.

[19] Byers K.A, Cox S.M, Lam R, Himsworth C.G (2019). “They're always there”:Resident experiences of living with rats in a disadvantaged urban neighborhood. BMC Public Health.

[20] Schulte P, Schwark L, Stassen P, Kouwenhoven T.J, Bornemann A, Speijer R.P (2013). Black shale formation during the Latest Danian Event and the Paleocene–Eocene Thermal Maximum in central Egypt:Two of a kind?. Palaeogeogr. Palaeoclimatol. Palaeoecol.

[21] Hoath R (2009). A Field Guide to the Mammals of Egypt.

[22] Khalil L.F, Jones A, Bray R.A (1994). Keys to the Cestode Parasites of Vertebrates.

[23] Saad A.I, Younis A.E, Rabei J.M (2018). Experimental life cycle of *Contracaecum*
*quadripapillatum* n. Sp. in white pelican (*Pelecanus*
*erythrorhynchus*) at Lake Nasser, Egypt:Morphological and genetic evidences. J. Egypt. Soc. Parasitol..

[24] Bowles J, McManus D.P (1993). Rapid discrimination of *Echinococcus* species and strains using a polymerase chain reaction-based RFLP method. Mol. Biochem. Parasitol..

[25] Wang C.R, Li L, Ni H.B, Zhai Y.Q, Chen A.H, Chen J, Zhu X.Q (2009). *Orientobilharzia**turkestanicum* is a member of *Schistosoma* genus based on phylogenetic analysis using ribosomal DNA sequences. Exp. Parasitol.

[26] Mariaux J (1998). A molecular phylogeny of the *Eucestoda*. J. Parasitol.

[27] Dereeper A, Audic S, Claverie J.M, Blanc G (2010). BLAST-EXPLORER helps you building datasets for phylogenetic analysis. BMC Evol. Biol..

[28] Owen D (1976). Cestodes in laboratory mice:Isolation of *Cataenotaenia*
*pusilla*. Lab. Anim..

[29] Younis D.A (2006). Some Studies on Parasites of Rats with Special Reference to These Transmissible to Man.

[30] Hughes R.C (1941). A key to the species of tapeworms in *Hymenolepis*. Trans. Am. Micros. Soc.

[31] Panti-May J.A, Servían A, Ferrari W, Zonta M.L, Hernández-Mena D.I, Hernández-Betancourt S.F, del Rosario Robles M, Machain-Williams C (2020). Morphological and molecular identification of hymenolepidid cestodes in children and synanthropic rodents from rural Mexico. Parasitol. Int..

[32] Hasanpour H, Najafi F, Gharagozlou M.J, Jafarpour Azami S, Fadavi A, Paknezhad N, Mowlavi G (2017). *Cysticercus fasciolaris* (*Taenia taeniaeformis* larval stage) in urban rats with illustration of histopathological changes in the liver. J. Med. Microbiol. Infect. Dis..

[33] Martínez M.L, Domínguez M.G, Morici G.E, Cavia R, de Oca D.P.M, Lovera R, Schapiro J.H, Caracostantogolo J.L (2013). Morphological and molecular identification of *Cysticercus fasciolaris* isolated from a rodent (*Rattus norvegicus*) from the province of Buenos Aires (Argentina). Rev. Argent. Microbiol..

[34] Lee B.W, Jeon B.S, Kim H.S, Kim H.C, Yoon B.I (2016). *Cysticercus fasciolaris* infection in wild rats (*Rattus norvegicus*) in Korea and formation of cysts by remodelling of collagen fibres. J. Vet. Diagn. Invest..

[35] Sharma R, Tiwari K, Birmingham K, Armstrong E, Montanez A, Guy R, Sepulveda Y, Mapp-Alexander V, DeAllie C (2017). *Cysticercus fasciolaris* in brown rats (*Rattus norvegicus*) in Grenada, West Indies. J. Parasitol. Res.

[36] Abdel Hamid M.M, Eljack I.A, Osman M.K.M, Elaagip A.H, Muneer M.S (2015). The prevalence of *Hymenolepis*
*nana* among preschool children of displacement communities in Khartoum state, Sudan:A cross-sectional study. Travel Med. Infect. Dis..

[37] Malsawmtluangi C, Prasad P.K, Biswal D.K, Tandon V (2011). Morphological and molecular identification of the metacestode parasitizing the liver of rodent hosts in bamboo growing areas of Mizoram, Northeast India. Bioinformation.

[38] Bowman D.D, Lynn R.C, Eberhard M.L (2014). Georgis'Parasitology for Veterinarians.

[39] Macnish M.G, Morgan-Ryan U.M, Monis P.T, Behnke J.M, Thompson R.C.A (2002). A molecular phylogeny of nuclear and mitochondrial sequences in *Hymenolepis nana* (*Cestoda*) supports the existence of a cryptic species. Parasitology.

[40] Olson P.D, Yoder K, Fajardo L.F, Marty A.M, van de Pas S, Olivier C, Relman D.A (2003). Lethal invasive cestodiasis in immunosuppressed patients. J. Infect. Dis..

[41] Shubber H.W.K, Nabeel M, Al-Tameemi M (2019). Comparative study morphological and molecular for infected rodent with two *Cestoda Hymenolepis nana* and *H. diminuta* in Al-Diwaniyah city, South of Iraq. J. Phys. Conf. Ser..

[42] Nkouawa A, Haukisalmi V, Li T, Nakao M, Lavikainen A, Chen X, Henttonen H, Ito A (2016). Cryptic diversity in hymenolepidid tapeworms infecting humans. Parasitol. Int..

[43] Hoggard K.R, Jarriel D.M, Bevelock T.J, Verocai G.G (2019). Prevalence survey of gastrointestinal and respiratory parasites of shelter cats in Northeastern Georgia, USA. Vet. Parasitol. Reg. Stud. Reports.

[44] Lavikainen A, Haukisalmi V, Lehtinen M.J, Henttonen H, Oksanen A, Meri S (2008). A phylogeny of members of the family *Taeniidae* based on the mitochondrial cox1 and nad1 gene data. Parasitology.

[45] Jia W, Yan H, Lou Z, Ni X, Dyachenko V, Li H, Littlewood D.T.J (2012). Mitochondrial genes and genomes support a cryptic species of tapeworm within *Taenia*
*taeniaeformis*. Acta Trop..

[46] Tijjani M, Majid R.A, Abdullahi S.A, Unyah N.Z (2020). Detection of rodent-borne parasitic pathogens of wild rats in Serdang, Selangor, Malaysia:A potential threat to human health. Int. J. Parasitol. Parasites Wildl.

[47] Panti-May J.A, Rodríguez-Vivas R.I, García-Prieto L, Servián A, Costa F (2020). Worldwide overview of human infections with *Hymenolepis diminuta*. Parasitol. Res.

[48] Bajer A, Alsarraf M, Dwużnik D, Mierzejewska E, Kołodziej-Sobocińska M, Jolanta Behnke-Borowczyk J, Banasiak L, Grzybek M, Tołkacz K, Kartawik N, Stańczak L, Opalińska P, Krokowska-Paluszak M, Górecki G, Alsarraf M, Behnke J (2020). Rodents as intermediate hosts of cestode parasites of mammalian carnivores and birds of prey in Poland, with the first data on the life-cycle of *Mesocestoides melesi*. Parasite Vectors.

[49] Dyab A.K, El-Salahy M.M, Abdelmoneiem H.M, Amin M.M, Mohammed M.F (2016). Parasitological studies on some intestinal parasites in primary school children in Aswan Governorate, Egypt. J. Egypt. Soc. Parasitol..

